# Dominant Allele Phylogeny and Constitutive Subgenome Haplotype Inference in Bananas Using Mitochondrial and Nuclear Markers

**DOI:** 10.1093/gbe/evx167

**Published:** 2017-08-27

**Authors:** Kariuki Samwel Muiruri, Anne Britt, Nelson Onzere Amugune, Edward Nguu, Simon Chan, Leena Tripathi

**Affiliations:** 1 International Institute of Tropical Agriculture (IITA), Nairobi, Kenya; 2 School of Biological Sciences, University of Nairobi, Kenya; 3 Department of Plant Biology, University of California, Davis; 4 Department of Biochemistry, University of Nairobi, Kenya

**Keywords:** banana, haplotype, genotypes, phylogeny, *NAD1* and *CENH3*

## Abstract

Cultivated bananas (*Musa* spp.) have undergone domestication patterns involving crosses of wild progenitors followed by long periods of clonal propagation. Majority of cultivated bananas are polyploids with different constitutive subgenomes and knowledge on phylogenies to their progenitors at the species and subspecies levels is essential. Here, the mitochondrial (*NAD1*) and nuclear (*CENH3*) markers were used to phylogenetically position cultivated banana genotypes to diploid progenitors. The *CENH3* nuclear marker was used to identify a minimum representative haplotype number in polyploids and diploid bananas based on single nucleotide polymorphisms. The mitochondrial marker *NAD1* was observed to be ideal in differentiating bananas of different genomic constitutions based on size of amplicons as well as sequence. The genotypes phylogenetically segregated based on the dominant genome; AAB genotypes grouped with AA and AAA, and the ABB together with BB. Both markers differentiated banana sections, but could not differentiate subspecies within the A genomic group. On the basis of *CENH3* marker, a total of 13 haplotypes (five in both diploid and triploid, three in diploids, and rest unique to triploids) were identified from the genotypes tested. The presence of haplotypes, which were common in diploids and triploids, stipulate possibility of a shared ancestry in the genotypes involved in this study. Furthermore, the presence of multiple haplotypes in some diploid bananas indicates their being heterozygous. The haplotypes identified in this study are of importance because they can be used to check the level of homozygozity in breeding lines as well as to track segregation in progenies.

## Introduction

Banana including plantain is among the world's most important staple food crops cultivated with an annual production of ∼145 million tonnes of which <17% is for export trade (FAOSTAT 2014). Africa contributes one-third of the global production with East Africa being the largest banana-growing region accounting for ∼40% of the total production in Africa. Banana production is hampered by pests and diseases including weevils and nematodes, fusarium wilt caused by the fungi *Fusarium oxysporum* f. sp. cubense, banana Xanthomonas wilt caused by bacteria *Xanthomonas campestris* pv*. musacearum*, black Sigatoka caused by *Mycosphaerella fijiensis* and viral diseases caused by *Banana streak virus* (BSV) and *Banana bunchy top virus* (BBTV; Jones 2000; Surridge etal. 2003; Tripathi etal. 2009). Large-scale producers can afford chemical control for the pests and diseases but the smallholder farmers, who produce at least 85% of the global total, cannot afford chemical control. The use of resistant cultivars is considered the most effective, economical and environmental friendly approach to control pests and diseases. Major banana breeding programs therefore target creation of pests and diseases resistant varieties by introgressing favorable characters from wild species and genotypes (Lorenzen etal. 2010). Breeding is however limited by narrow genetic diversity and the sterility of the polyploid genomes of cultivated banana (Lorenzen etal. 2010). This has resulted in renewed interest in cultivated and noncultivated bananas and led to an increase of interest regarding their evolutionary relationships (Christelová etal. 2011; Häkkinen 2013; Hřibová etal. 2011; Boonruangrod etal. 2008; Baurens etal. 2010).

The genus *Musa* has for a long time been classified into five sections, namely *Eumusa* (*n* = 11), *Rhodochlamys* (*n* = 11), *Callimusa* (*n* = 10 or *n* = 9), *Australimusa* (*n* = 10; Cheesman 1950), and *Ingentimusa* (*n* = 7; Argent 1976), with majority of cultivated bananas being grouped in *Eumusa*. However, recent studies based on molecular phylogenetics have found no support for the five sections and these have now been reappraised and collapsed to only two; section *Musa* (encompassing the former sections *Rhodochlamys* and *Eumusa*) and *Callimusa* (with the former *Callimusa*, *Australimusa*, and *Ingentimusa*; Häkkinen 2013). However, because the reappraisal efforts by Häkkinen was a proposition, the old sections will be maintained in this study, but inconsistencies and similarities (to the old and new sections) will be highlighted whenever they arise. There are four progenitors of the domesticated banana *Musa acuminata* Colla (A genome, *2n = 22*), *Musa balbisiana* Colla (B genome, *2n = 22*), and to a lesser extent, *Musa schizocarpa* (S genome) and *Musa textilis/Musa maclayi* (T genome; Cheesman 1950). On the basis of chloroplast and mitochondrial genomes analysis, the species of *M. acuminata* have been classified into eight subspecies (subsp.), including subsp. *banksii* from Papua New Guinea, subsp. *errans* from Philippines, subsp. *zebrina* from the Indonesia, subsp. *malaccensis*, subsp. *microcarpa*, subsp. *burmannicoides*, subsp. *burmannica* and subsp. *siamea* with a distribution in Thailand, Malaysia, and Philippines (Carreel etal. 2002)*.* No subspecies have been identified in *M. balbisiana* until now*.* Cultivated *Musa* clones are mostly derived from intersubspecific crossing of *M. acuminata* (A genome) and to a lesser extent interspecific hybrids of *M. acuminata* × *M. balbisiana* (B genome) although there still exists a few other hybrids from *M. acuminata* × *M. schizocarpa* (S genome) and *M. acuminata* × *M. textilis* (T genome) crosses respectively (Hřibová etal. 2011). The haploid genome contributed by *M. acuminata* is referred to as the A genome and that from *M. balbisiana* the B genome. On the basis of morphological characters and genetic composition, cultivated bananas were grouped into the genotypes AA, AB, AAA, AAB, ABB (Simmonds and Shepherd 1955). The majority of economically important banana cultivars belongs to the AAA genotype, which is characterized by low genetic diversity due to clonal propagation of nearly-sterile dominant cultivars (Baurens etal. 2010).

Cultivated banana and plantain commonly exist as autopolyploids and allopolyploids, the later representing different combinations of A and B and sometimes S and T genomes. Mutations may exist in heterozygous allele sequences within individual subgenomes as well as in homoeologous sequences among the polyploid’s constitutive subgenomes (Kaur etal. 2012). Identification of number of gene-alleles present in polyploids is very important as variability in numbers has been observed to have either additive or nonadditive expression patterns. Furthermore, gene redundancy in polyploid bananas covers-up for loss-of-function or even rescue lethal mutations arising in the homeologs and this masks evolutionary changes because they are not morphologically visible. Moreover, allele-dosage can help in identification of haplotypes which can in turn help identify progenitors contributing to the polyploid genome (Baurens etal. 2010; Boonruangrod etal. 2008; Li etal. 2013). The variations due to subgenomic alleles may sometimes be difficult to identify without mapping of the polymorphic loci, clone-based-sequencing or use of the latest approaches of haplotyping like genotyping by sequencing (GBS). Moreover, some of mapping approaches like Simple Sequence Repeats (SSRs) may not clearly identify biases like segregation bias common in mapping populations. Furthermore, most subgenomic allele discriminatory methods are expensive and out of reach for majority of laboratories especially those in developing countries. Moreover, the approaches used in haplotype identification in banana have included manually comparing and scoring amplified bands on agarose gel (Boonruangrod etal. 2008) which is laborious and could result in bias. Despite the advancement in haplotype identification techniques like next generation sequencing, the prices are still prohibitive for them to be used in some of the laboratories. Low cost approaches that simplify the identification of progenitors from cultivated bananas would contribute more to banana breeding efforts.

Amplified sequences have previously been used to identify homologs, homeologs, SNP-alleles as well as haplotypes in several crops like maize (Ching etal. 2002; Ding and Cantor 2003). The use of amplified sequences to identify haplotypes has led to development of algorithms that enable phasing (Neigenfind etal. 2008; Browning and Browning 2011; Efros and Halperin 2012; Guo etal. 2012). As DNA in polyploids contains multiple nucleotide calls at regions in which the constitutive genomes differ, these can be used to identify haplotypes of a specific gene within the polyploid genomes. Haplotypes especially from low-copy genes like *CENH3* can be used to identify diploid progenitors in polyploids as well as for screening introgression lines (IL) in breeding. The use of amplified sequence drastically reduces haplotyping cost and especially for direct sequencing of PCR products compared with the long procedure of haplotyping based on cloning.

This study used the partial coding sequence of the markers *centromere specific histone 3* (*CENH3*) and *nicotinamide adenine dinucleotide dehydrogenase subunit 1* (*NAD1*) to establish the relationships between cultivated diploid and triploid as well as wild-type diploid progenitor bananas. On the basis of the nuclear gene *CENH3*, we further inferred haplotype(s) from amplified sequence of triploid and diploid bananas. The mitochondrial gene (*NAD1*) coding for NADH dehydrogenase subunit 1, an enzyme in metabolism of fatty acids, sugar and amino acids is generally conserved but variable enough to be used in differentiating taxonomic levels in plant molecular phylogeny (Bremer etal. 2002). The *CENH3* gene is functionally conserved but is highly variable within the N-terminal tail region and exists in single or multiple copies in plants (Dunemann etal. 2014; Hirsch etal. 2009; Masonbrink etal. 2014; Wang etal. 2011). In this study, we obtained comparative phylogeny as well as representative haplotypes in bananas using the mitochondrial and nuclear genes *NAD1* and *CENH3* respectively.

## Materials and Methods

### Plant Material

In this study, a total of 40 banana genotypes/cultivars with variable genomic compositions were used (table 1). The samples included 10 genotypes of AA, 6 of AAA, 7 of AAB, 2 of AB, 6 of ABB, 1 of AS, and 5 of BB genomic composition and one tetraploid (ABBT cultivar) and the species *Musa ornata* and *M. textilis* from the sections *Rhodochlamys* and *Australimusa*, respectively. The cultivar “Ngombe” was obtained from Kenyan Agricultural and Livestock Research Organization (KALRO) and initiated as the invitro culture at IITA-Kenya. The remaining samples were obtained from Bioversity International *Musa* International Transit Center (ITC) hosted in KU Leuven, Belgium (Ruas etal. 2017).

**Table 1:** Banana Species, Genotypes and Cultivars Used in the Phylogeny and Haplotype Inference

No.	Accession No.	Accession Name	Section	Species/Group	Subspecies/Subgroup	Country of Origin
1	ITC0046	Monthan	Eumusa	ABB	subgr. Monthan	India
2	ITC0245	Safet Velchi	Eumusa	AB	subgr. Ney Poovan	India
3	ITC0247	Honduras	Eumusa	*balbisiana*	n/a	Honduras
4	ITC0420	Pisang Kayu	Eumusa	AAA	subgr. Orotava	Indonesia (IDN 098)
5	ITC0472	Pelipita	Eumusa	ABB	n/a	Philippines
6	ITC0582	Lady Finger	Eumusa	AAB	subgr. Nadan	India
7	ITC0637	*Musa ornata*	Rhodochlamys	*ornata*	subsp. *ornata*	–
8	ITC0659	Namwa Khom	Eumusa	ABB	subgr. Pisang Awak	Thailand (THA 011)
9	ITC0660	Khae Phrae	Eumusa	*acuminata*	subsp. *siamea*	Thailand (THA 015)
10	ITC0767	Dole	Eumusa	ABB	subgr. Bluggoe	–
11	ITC1028	Agutay	Eumusa	*acuminata*	n/a	Philippines
12	ITC1034	Kunnan	Eumusa	AB	subgr. Ney Poovan	India, Kerala
13	ITC1064	Pisang Bakar	Eumusa	AAA	subgr. Ambon	Indonesia (IDN 106)
14	ITC1120	Tani	Eumusa	*balbisiana*	n/a	–
15	ITC1138	Saba	Eumusa	ABB	subgr. Saba	Philippines
16	ITC1140	Red Yade	Eumusa	AAB	subgr. Plantain	Cameroon
17	ITC1152	Wompa	Eumusa	AS	n/a	Papua New Guinea (PNG 063)
18	ITC1156	Pisang Batu	Eumusa	*balbisiana*	n/a	Indonesia (IDN 080)
19	ITC1177	Zebrina	Eumusa	*acuminata*	subsp. *zebrina*	Indonesia
20	ITC1187	Tomolo	Eumusa	AA	n/a	Papua New Guinea (PNG023)
21	ITC0393	Truncata	Eumusa	*acuminata*	subsp. *truncata*	–
22	ITC0084	Mbwazirume	Eumusa	AAA	subgr. Mutika/Lujugira	Burundi
23	ITC0652	Kluai Tiparot	Eumusa	ABB	n/a	Thailand (THA 020)
24	ITC0769	Figue Pomme Géante	Eumusa	AAB	subgr. Silk	Guadeloupe
25	ITC1325	Orishele	Eumusa	AAB	subgr. Plantain	Nigeria
26	ITC0623	Banksii	Eumusa	*acuminata*	subsp. *banksii*	Papua New Guinea
27	ITC0249	Calcutta 4	Eumusa	*acuminata*	subsp. *burmannicoides*	India
28	ITC0653	Pisang Mas	Eumusa	AA	subgr. Sucrier	Malaysia
29	ITC0962	Prata Ana	Eumusa	AAB	subgr. Pome	Brazil
30	ITC1441	Pisang Ceylan	Eumusa	AAB	subgr. Mysore	Malaysia
31	ITC0250	Malaccensis	Eumusa	*acuminata*	subsp. *malaccensis*	–
32	ITC0093	Long Tavoy	Eumusa	*acuminata*	subsp. *burmannica*	–
33	ITC1511	DH Pahang (CIRAD 930)	Eumusa	*acuminata*	subsp. *malaccensis*	Guadeloupe
34	ITC0649	Foconah	Eumusa	AAB	–	Pome/Prata
35	–	Ngombe	Eumusa	AAA	East African Highland Banana	Kenya
36	ITC0180	Grande Naine	Eumusa	AAA	Cavendish	ITC
37	ITC1588	Lal Velchi	Eumusa	*balbisiana*	type 3	ITC
38	ITC1063	Pisang Klutuk Wulung	Eumusa	*balbisiana*	type 4	Indonesia (IDN 056)
39	ITC1072	Textilis	Australimusa	*textilis*	subsp. *textilis*	–
40	ITC1238	Yawa 2	Tetraploid	ABBT	n/a	Papua New Guinea

### Genomic DNA Extraction

Genomic DNA was extracted from all the cultivars using CTAB (Porebski etal. 1997) except for the cultivar “Ngombe” that was extracted using Qiagen DNA extraction kit (Qiagen, Hilden, Germany).

### Primer Design

Primers were designed to amplify partial coding sequence of *CENH3* and *NAD1* genes. Primers for *CENH3* were designed based on coding sequence of “Doubled Haploid Pahang” (DH Pahang) using PrimerSelect-DNASTAR software (Madison, USA). The *CENH3* primers were designed to target a 322 bp amplicon from position 6211 to 6533, which corresponds to the exon 1 and part of intron 1 obtained from in silico analysis of the 7 kb genomic sequence from *M. acuminata* genotype “DH Pahang”.

Sequences representing 27 species for *NAD1* within the monocots group were downloaded from the National Centre for Biotechnology Information (NCBI; http://www.ncbi.nlm.nih.gov) GenBank release 186 and further assembled in Sequencher version 4.1 (Ann Arbor, MI) (supplementary table 1, Supplementary Material online). The positions of variable and conserved regions were noted within the consensus sequence, which was then exported for primer design. For *NAD1*, positions 215–231 for the forward and 1598–1615 for the reverse primers out of the 1,726 bp long sequence were targeted. The best primer pairs were selected for each target region and synthesized from Inqaba Biotech (Pretoria, SA) and listed in table 2.

**Table 2:** List of Primers Used in This Study

	Primer Name	Primer Sequence
1	CENH3_FL_1F	CCCACCTCTTTTGTTTTCTTG
2	CENH3_FL_RI2	TTTTAGGGGATGTTACGGTTAGAC
3	CENH3_gene_F2	CTGCTGTGATGGCGAGAAC
4	CENH3_gene_R2	CTGGTGGCCGTGGTTC
5	CENH3_gene_R4	TGAACCGTCCCATAATACC
6	CENH3_gene_F4	GGTTGGCCACTGAAGATAC
7	CENH3_gene_F11	GGTGAAAACAAGCCAAACTA
8	CENH3_gene_R11	ACGCAAAAGATTAATTATGTATGT
9	NAD1_c10F	CACGTTGCTTTCTACCACATCG
10	NAD1_c10R	CCCCTACTACTACTCATTACTC
11	NAD1_c5F	GTCCCCGGCCAGAACCAC
12	NAD1_c5R	GCAGTCCGGGGCACAAG
13	NAD1_intr2_2F	CGTCGCAAGGCTCATTTTTAG
14	NAD1_intr2_2R	CGCCCGCCCTTTATTTAGATG
15	NAD1_int2_F	GCGCCCGCCCTTTATTTAGAT
16	NAD1_int2_R	GCGCCCGCCCTTTATTTAGAT

### PCR Analysis, Sequencing and Sequence Assembly

Six primer sets (three for each target region) were first optimized with eight genotypes selected from the 40 samples under study. On the basis of quality of PCR products and variability in the sequences, the best primer pair for each marker was selected and used to amplify the remaining samples. The sequences of the two primer pairs were: *CENH3*_gene_F2: CTGCTGTGATGGCGAGAAC (6530–6548) and *CENH3*_gene_R2: CTGGTGGCCGTGGTTC (6212–6227) for amplification of *CENH3* gene and *NAD1*_c5F: GTCCCCGGCCAGAACCAC and *NAD1*_c5R: GCAGTCCGGGGCACAAG for amplification of *NAD1* gene*.* PCR reactions were performed in a final volume of 20 µl reaction using the Bioneer PCR premix (Bioneer, Daejeon, South Korea) containing: 1 U of *Top* DNA polymerase, 250 µM dNTP, 10 mM Tris–HCl (pH 9.0), 30 mM KCl and 1.5 mM MgCl_2_. The amplifications were done in Applied Biosystem (ABI) GeneAmp PCR machine 9,700 using the profile: Initial denaturation of 94 °C for 5 min, 40 cycles of 94 °C for 30 s, annealing temperature of 59 °C for 1 min, 72 °C extension for 40 s, and a final extension of 72 °C for 10 min for *CENH3* gene, while *NAD1* was amplified with an initial denaturation of 94 °C for 5 min, 40 cycles of 94 °C for 30 s, annealing temperature of at 64 °C for 30 s and extended at 72 °C for 1.5 min. The PCR products were separated on a 1.5% agarose gel stained with gelred (Biotium, CA) to confirm successful amplifications. Products with successful amplifications were cleaned using Bioneer PCR purification kit (Bioneer, Daejeon, South Korea) according to the manufacturer's protocol.

Sequencing of PCR products was performed from both ends (Forward and reverse) using automated sequencers ABI 3130 and 3730 prism as per set protocols. Sequences were assembled and edited in Sequencher version 4.1. All ends that had low confidence base calls were trimmed to retain high confidence sequences. Sequence assembly parameters were set at 85 and 25 and assembly done using the “assemble automatically parameter”. The criteria for calling heterozygous secondary bases were set at 30% of the primary chromatogram. Finally, IUPAC codes were assigned to all the positions that were meeting the criteria for multiple calls and sequences exported in FASTA concatenated format for further analyses.

### Sequence Alignment and Phylogenetic Reconstruction

FASTA concatenated sequences were aligned in ClustalW (Thompson etal. 1994) as implemented in MEGA5 (Tamura etal. 2011). Alignment of *CENH3* sequences was done default Gap Opening Penalty (GOP), Gap Extension Penalty (GEP) and DNA weight matrix for both markers. Phylogeny reconstruction was performed by maximum likelihood (ML) for both markers. The two markers had different parameters in reconstruction except for the bootstrap (BP) statistical support of individual clades and the respective number of BP replicates which was set at 1,000. Model selection was performed in phyML (Guindon etal. 2010). During phylogenetic analysis for *CENH3* sequences, the rates among sites were gamma distributed with a gamma parameter of 8.0, gaps with missing sequence sections were treated by pairwise deletion for *CENH3*. Phylogenetic reconstruction for *NAD1* used the model Kimura 2 parameter (KC) and the rates among sites were uniform while gaps and missing data were treated by complete deletion. Equally for *NAD1* gaps in the alignment gaps were treated as missing data.

### Haplotype Inference

Single nucleotide polymorphisms (SNPs) for *CENH3* to which had been assigned IUPAC codes were exported to excel from MEGA5 and saved as a .cvs file. The SNP positions were grouped as per the genotype’s ploidy (triploids and diploids). Triploid genotype that only had two nucleotide calls (a primary and secondary) at the target SNP site (missing a tertiary chromatogram) the IUPAC code N (A or T or G or C) was added in place of the third, indicating that the third base call may have been any of the four at that position (including homozygous type). SNPs within the diploid genotypes that only had one base call equally had an N added to complete the two base calls associated with diploidy (supplementary table 2, Supplementary Material online). The species *M. ornata* and *M. textilis* plus any other genotype that did not have multiple calls were assumed to be homozygous and therefore omitted from the analysis.

The software SATlotyper (Neigenfind etal. 2008) was used for haplotype inference. Both triploid and diploid SNP positions were run with the SATsolvers set as MiniSat_v1.14_cygwin. All the SNP positions having multiple calls were used for analysis with a BP value of 100. Haplotypes identified in haploids were physically checked for duplication in triploids. Haplotypes that were duplicated in diploid and triploids were treated as a single incidence. Duplicated haplotypes together with the unique ones (occurring only once in either diploids or triploids) were considered specific to both diploids and triploids. All haplotypes and the genotypes from which they were inferred were tabulated to indicate relatedness.

## Results

### PCR Amplification of *CENH3* and *NAD1* Genes in Banana Genotypes

**Fig. 1.**
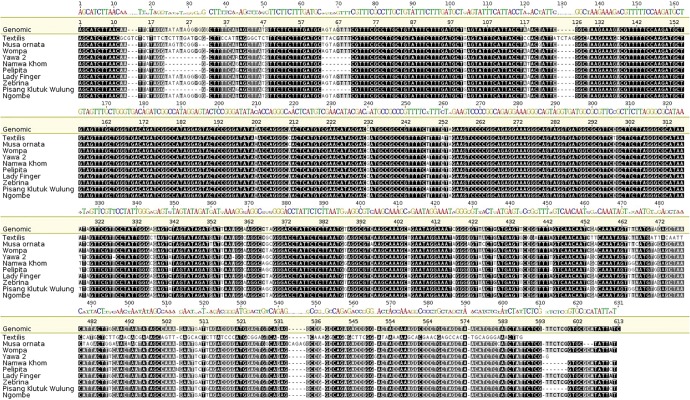
—Multiple sequence alignment of *NAD1* sequences representing various genotypes and species from the different genomic groups. The genotypes included are Wompa (AS), Yawa 2 (ABBT), Namwa Khom (ABB), Pelipita (ABB), Lady Finger (AAB), Zebrina (AA), Pisang Klutuk Wulung (BB) and Ngombe (AAA), and species *Musa textilis* (T-genome) and *Musa ornata.* The reference genomic sequence represents the partial genome region from the *Musa acuminata* genome GenBank number GCAIC01015283.

Amplification was performed on banana genotypes with different genomic compositions and different sizes of amplicons were obtained using *NAD1* marker (supplementary fig. 1, Supplementary Material online). However, a single amplicon of ∼322 bp was obtained with the marker *CENH3*. On the basis of *NAD1* specific primers, bananas of genomic groups AAA, AA, AAB, and AB were observed to have a band size of ∼1,000 bp, whereas ABB and BB genomic groups had a band size of 1,500 bp. The species *M. textilis* (T-genome) was observed to have the smallest band size of ∼900 bp. None of the genotype used in this study showed multiple bands on the gel. PCR products were sequenced in order to identify the dominant allele and also to check reasons behind the differences in size of *NAD1* amplicons. To ensure sequence authenticity, sequencing was done bidirectionally using the same primers used for amplification (table 2). The differences in sizes of bands in amplicons from different genomic groups observed in PCR were identified to be due to insertions and deletions (indels; fig. 1).

### Sectional and Genomic Group Congruence

**Fig. 2.**
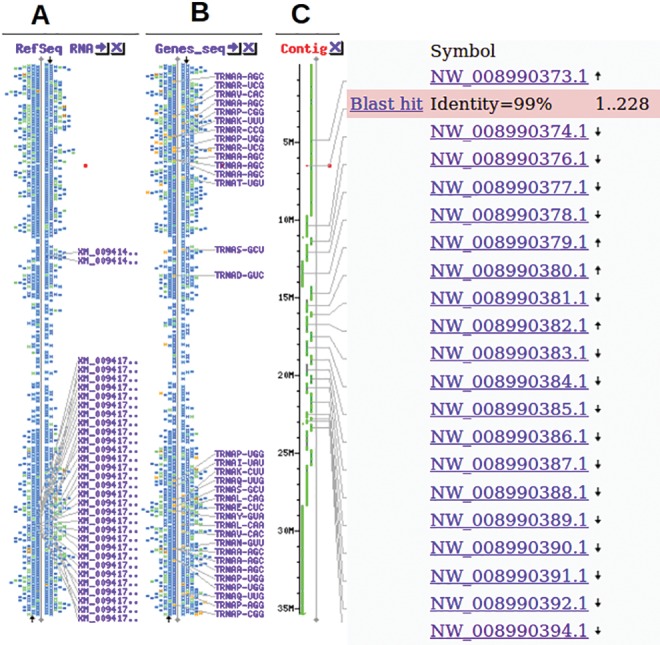
—Banana *CENH3* sequence mapped in context of the *Musa acuminata* reference whole genome contig sequences (WGS), reference sequences (RefSeq), and gene sequences (Gene_Seq). A-RefSeq RNA map, B-Genes_seq map, and C-WGS contig map.

Bidirectional sequencing and clean-up resulted in a total of 59 sequences, 36 for *NAD1* and 23 for *CENH3*. The data for the genotypes Pelipita, Calcutta 4, Tani, Red Yade, and Yawa 2 were treated as missing data for the *NAD1* marker due to consistently poor quality of sequence. The sequences obtained were deposited in the NCBI under accession numbers KP751256–KP751292 for *NAD1* and KP751293–KP751328 for *CENH3.* Because *CENH3* was used in haplotype inference, it was paramount to map it in the context of the available banana whole genome (fig. 2). The *CENH3* gene was observed to be within the contig NW_008990373.1 of the *M. acuminata* subsp. *malaccensis* chromosome 8 genomic scaffold (Ma08_p09050.1; banana genome, annotation version 2) specifically within scaffold ASM31385v2_scafold_4 (D’Hont etal. 2012) of the NCBI annotation release 101 (fig. 2).

Multiple alignments of clean sequences of *NAD1* and *CENH3* for all the 40 genotypes resulted in an aligned length of 1,448 bp (including indels in some cultivars) and 278 bp for the two markers respectively. Among all sequences, 196 and four SNPs were observed to be variable for *NAD1* and *CENH3*, respectively. In order to ensure that the observed SNP variations were genuine and not due to PCR errors, they were further confirmed by resequencing (of independent PCR products) in the genotypes involved. The best ML model selected for the markers *CENH3* and *NAD1* were TN93 (supplementary table 3, Supplementary Material online) and GTR (supplementary table 4, Supplementary Material online), respectively. During model selection, the two markers were observed to have different evolutionary rates and hence alignments were conducted separately. Twenty SNP positions in *NAD1* alignment were observed to be sufficient to differentiate A and B genomes of banana (table 3).

**Table 3:** Variable SNPs from the Marker *NAD1* that Differentiated A and B Banana Genomes

		SNP Number																			
Genotype	Genomic Group	1	2	3	4	5	6	7	8	9	10	11	12	13	14	15	16	17	18	19	20
Dole	ABB	C	C	G	T	A	C	A	T	T	A	C	G	C	T	G	G	A	C	T	C
Honduras	BB	C	C	G	T	A	C	A	T	T	A	C	G	C	T	G	G	A	C	T	C
Kluai Tiparot	ABB	C	C	G	T	A	C	A	T	T	A	C	G	C	T	G	G	A	C	T	C
Monthan	ABB	C	C	G	T	A	C	A	T	T	A	C	G	C	T	G	G	A	C	T	C
Pelipita	ABB	C	C	G	T	A	C	A	T	T	A	C	G	C	T	G	G	A	C	T	C
Pisang Batu	BB	C	C	G	T	A	C	A	T	T	A	C	G	C	T	G	G	A	C	T	C
Pisang Klutuk Wulung	BB	C	C	G	T	A	C	A	T	T	A	C	G	C	T	G	G	A	C	T	C
Tani	BB	C	C	G	T	A	C	A	T	T	A	C	G	C	T	G	G	A	C	T	C
Figue Pomme Geante	AAB	A	A	T	G	T	A	C	A	G	T	A	T	A	G	T	T	T	T	A	G
Lady Finger	AAB	A	A	T	G	T	A	C	A	G	T	A	T	A	G	T	T	T	T	A	G
*Musa acuminata* spp. *banksii*	AA	A	A	T	G	T	A	C	A	G	T	A	T	A	G	T	T	T	T	A	G
Calcutta 4	AA	A	A	T	G	T	A	C	A	G	T	A	T	A	G	T	T	T	T	A	G
*Musa acuminata* spp.*malaccensis*	AA	A	A	T	G	T	A	C	A	G	T	A	T	A	G	T	T	T	T	A	G
*Musa acuminata* spp. *truncata*	AA	C	C	T	G	T	A	C	A	G	A	A	T	A	G	T	T	T	T	A	G
Wompa	AS	A	A	T	G	T	A	C	A	G	T	A	T	A	G	T	T	T	T	A	G
Zebrina	AA	A	A	T	G	T	A	C	A	G	T	A	T	A	G	T	T	T	T	A	G

A, adenine; C, cytosine; G, guanine; T, thymine; greyed cells represent AA, AS, AAA, and AAB genotypes while white ones represent ABB and BB genotypes.

**Table 4:** Haplotypes Inferred from Diploid and Polyploidy Bananas Based on *CENH3* Marker

Haplotype number	Haplotype	Genotypes^b^
1	CGAAACT^a^	3, 6, 10, 12, 15, 18, 23, 37, 38
2	CGAAATC^a^	4, 5, 6, 9, 11, 19, 24, 25, 26, 29, 32, 33, 34
3	CGAAGTC^a^	17, 20, 22, 35
4	CGATATC^a^	15, 38
5	CGGAATC^a^	2, 20, 29, 30
6	CGAAGTT	17
7	CGGAGCC	19
8	GCAAATC	2
9	CGGAGTC	30
10	GCAAATT	29
11	GCAATCG	16
12	GGGAATC	8
13	GGGAATT	8

The column genotypes indicate the genotypes that had representation within that haplotype. The genotype numbers are as listed in the last column.

a Haplotypes that were present in both diploids and triploids.

b Numbers in the genotype column represent the names as listed in table 1.

### Genotype Congruence Based on the Marker *NAD1*

**Fig. 3.**
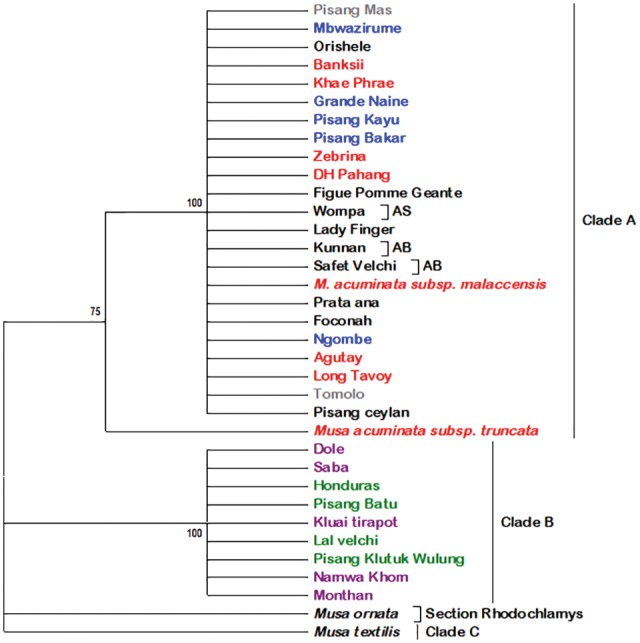
—Phylogenetic tree based on the marker *NAD1.* Numbers above branches represent the bootstrap (BP) support values. Genotypes in blue are triploid AAA, gray represent cultivated diploid AA, red represent *M. acuminata* wild type relatives, green represent wild type BB, purple are ABB genotypes while names of genotypes in black represent AAB except those that have a bracket and description after. The genotypes in black and with letters after interspecific diploids and have the genome composition indicated after the brackets.

Three clades were obtained on phylogenetic reconstruction with *NAD1*, and denoted as clades A, B and C (fig. 3 and supplementary data 1, Supplementary Material online). The clades were strongly supported at 75 and 100 BP values for clades A and B, respectively. The clade A was represented by triploid and diploid genotypes from different genomic groups. The triploid genotypes clustered in this clade were either AAA or AAB genomic groups (fig. 3). Diploids in this clade have AA genomic composition, AS genomic constitution represented by the genotype Wompa, and AB genotypes represented by Safet Velchi and Kunnan (fig. 3). *NAD1* marker was unable to differentiate diploids within AA genome represented by the subspecies *banksii*, *zebrina*, *malaccensis*, and *siamea*. However, the diploid AA in the subspecies *truncata* segregated within clade A albeit as a subclade.

The clade B in the *NAD1* phylogeny had representation from BB and ABB genomic groups and was strongly supported at a BP support of 100. This clade had nine subclades represented by individual genotypes of both BB and ABB genomic constitution. The genotypes with ABB genome within this clade were represented by Saba, Namwa Khom, Kluai Tiparot, Monthan, and Dole (fig. 3). The genotypes with BB genomes within this clade were represented by Pisang Klutuk Wulung, Pisang Batu, Lal Velchi, and Honduras.

In the third clade C had the species *M. textilis* as the only representative of the section *Australimusa*. The species *M. ornata* of the section Rhodochlamys did not cluster to any of the three clades (A, B or C).

### Genotype Congruence Based on the Marker *CENH3*

**Fig. 4.**
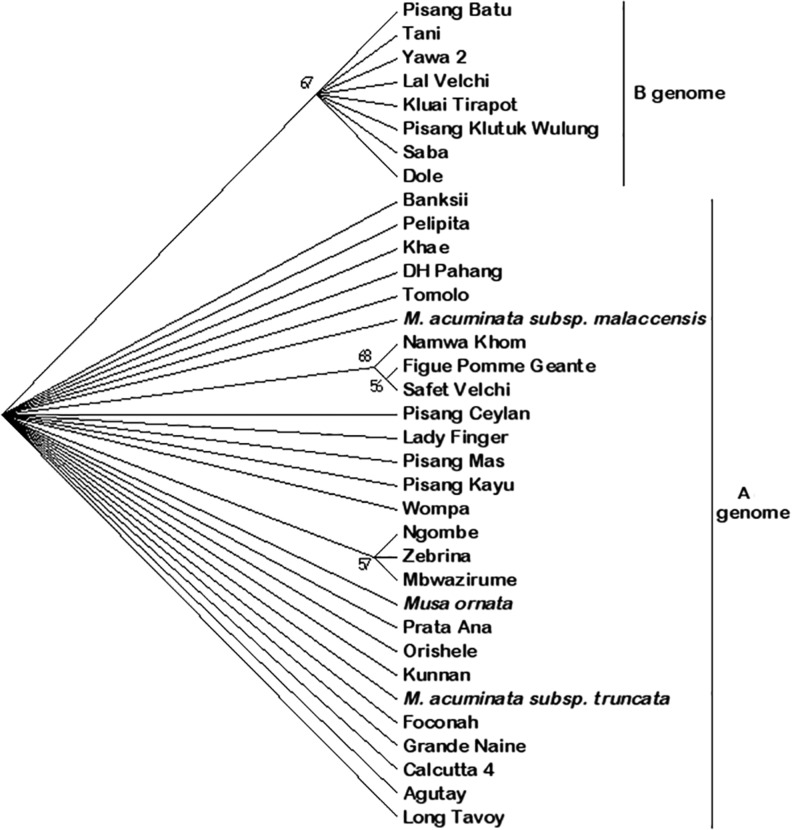
—Phylogenetic positioning of different banana genotypes based on the marker *CENH3*. The numbers on top of each clade represent bootstrap (BP) support values.

The phylogeny based on the marker *CENH3* had two major clades and associated subclades (fig. 4 and supplementary data 2, Supplementary Material online). The first clade the “A genome genotypes” had representative genotypes with A genome (AA, AB, AAA, and AAB) except for the genotype Wompa, which is an interspecific hybrid between *M. acuminata* and *M. schizocarpa* (AS genomic composition) and the species *M. ornata*. This marker was able to differentiate subspecies and genome groups within the A genome clade and these appeared in the phylogenetic tree as subclades, each having one or more genotype(s). Two subclades with more than one genotype were observed to segregate from the A genome clade. These two multigenotype subclades included the one with the genotypes Zebrina (AA), Mbwazirume (AAA), and Ngombe (AAA), which were supported at a 57 BP. The second subclade within this clade that had multiple genotypes was supported at 68 BP value and had the genotypes Figue Pomme Géante (AAB), Safet Velchi (AB), and Namwa Khom (ABB). This clade also had multiple single-genotype subclades with different genomic groups (fig. 4). The single genotype clades included those with the genotypes, Banksii, Pelipita, Khae Phrae, DH Pahang, Tomolo, *Musa accuminata* subsp. *malaccensis*, Pisang Ceylan, Lady Finger, Pisang Mas, Pisang Kayu, Wompa, *M. ornata*, Prata Ana, Orishele, Kunnan, *Musa accuminata* subsp. *truncata*, Foconah, Grande naine, Calcutta 4, Agutay, and Long Tavoy (fig. 4).

The second clade based on *CENH3* marker, “B genome clade” had genotypes from the BB, ABB, and ABBT genomic groups (fig. 4). This clade had a total of eight genotypes, out of which four were from BB, three from ABB and one with ABBT genomic composition. The BB genotypes in this subclade included the genotypes Pisang Batu, Lal Velchi, Pisang Klutuk Wulung, and Tani. The ABB genotypes in this B genome clade included Saba, Dole and Kluai Tirapot. The only tetraploid within this clade was represented by Yawa 2 (ABBT). The genotypes Honduras, Monthan, Pisang Bakar and *M. textilis* were treated as missing data resulting from failure to PCR amplify.

### Haplotypes in Diploids and Triploids

Haplotypes were obtained by analyzing SNP positions with multiple base calls within the *CENH3* gene. Seven SNP positions in 34 genotypes were used to infer haplotypes (table 4). A total of 13 haplotypes were observed based on the seven SNPs and out of these five were found to be present in diploids and triploids, three SNPs were observed in diploids only while the rest were unique to triploids. Haplotype numbers 1, 2, 3, 5 were observed to have the highest genotype representation (table 4).

The genotypes [numbers represent the order of names as listed in table 1 were Pisang Batu (18), Honduras (3), Pisang Klutuk Wulung (38), and Lal Velchi (37)] with B genome were represented within haplotype number 1 (table 4). The genotype Lady Finger (6) with the genomic composition AAB, the genotypes Dole (10), Kluai Tiparot (23), and Saba (15) from ABB as well as Kunnan (12) from AB genomic group were also observed to be associated with this haplotype. Haplotype number 2 has representative from diploids and triploids with A genome as well as interspecific triploids with A and B genome. The diploids with AA genomic group associating with haplotype 2 were represented by the genotypes Agutay (11), Banksii (26), Khae Phrae (9), DH Pahang (33), Zebrina (19), and Long Tavoy (32). The triploid AAA linking to this haplotype was represented by the genotype Pisang Kayu (4). Moreover, haplotype 2 had genotypes with AAB and ABB genomic composition closely linking to it at these seven SNP positions. The genotypes Figue Pomme Géante (24), Prata Ana (29), Orishele (25), Foconah (34), and Lady Finger (6) were the AAB genomic group genotypes that were closely linked to haplotype 2. The genotype Pelipita (5) was the only genotype with the ABB genomic composition that had close sequence similarity to this haplotype. Haplotype number 3 had mainly A genome containing genotypes Mbwazirume (22), Tomolo (20), Wompa (17), and Ngombe (35). Haplotype number 4 was closely associated with two genotypes Pisang Klutuk Wulung (38) and Saba (15). Pisang Klutuk Wulung is a BB genomic group genotype, while the genotype Saba is of ABB genomic composition. The genotypes Pisang Ceylan (30), Tomolo (20), Prata Ana (29), and Safet Velchi (2) were the genotypes that were within haplotype 5. Haplotypes 6–13 had only one representative genotype, Wompa (17), Zebrina (19), Safet Velchi (2), Pisang Ceylan (30), Prata Ana (29), Red Yade (16), and Namwa Khom (8) for haplotypes 6–13 respectively (table 4).

## Discussion

Several studies have been conducted using molecular approaches in banana to identify their origins and domestication patterns especially cultivated polyploids from their diploid progenitors (De Langhe etal. 2010; Hippolyte etal. 2012; Lescot etal. 2008). In this study, a mitochondrial and a nuclear marker were used to establish the phylogeny and specific haplotypes of cultivated triploids and a tetraploid banana to the potential diploid progenitors. The phylogenies using the dominant allele with both markers resulted in clear segregation of genotypes based on the respective genomic constitution. The major genome was observed to dominate with AAB genotypes segregating together with AA and AAA genotypes and ABB with BB genotypes. Moreover, the A genome was observed to dominate in the genotypes with AB and AS genomic composition as they were observed to segregate with the AA and AAA. It is therefore important to consider the subgenomic alleles through haplotyping.

The sizes of *NAD1* amplicons were dependent on the genomic group, which was attributable to insertions and deletions (indels) within the sequences. The genotypes with BB and ABB genomes were observed to have a larger amplicon in comparison with genotypes with AA, AAB, and AAA genomic composition. This marker was able to differentiate the B cytoplasm in ABB genotypes and A cytoplasm in AAB heterogenomic triploids, as clearly reflected through the band sizes of amplicons (supplementary fig. 1, Supplementary Material online).

Three different sections (*Eumusa*, *Rhodochlamys*, and *Australimusa*) represented in this work were differentiated using the two markers. The result that *M. ornata* representative of the section *Rhodochlamys* clustered with the A genome genotypes of the section *Eumusa* using the *CenH3* marker is consistent with other studies (Christelová etal. 2011; Hřibová etal. 2011; Wong 2002). This observation corroborates Wong’s (2002) and Christelová etal.’s (2011) findings and suggestions that these two sections should be merged, as there are no great genetic differences that warrant their positioning into different sections.

The only representative of the AS diploid genotype “Wompa” belonging to *M. schizocarpa* was observed to cluster with the *M. acuminata* clade within the section *Eumusa* hence supporting the point made by Hřibová etal. (2011) that*M. schizocarpa* is closely related to *M. acuminata.* Alternatively, this clustering could also be due to the A-allele in this AS genotype being the dominant allele and therefore easily amplifiable and detectable.

It was observed that the B genome clade and the respective genotypes are distant to that of *M. acuminata* despite being in the same section (figs. 3 and 4). Despite being in a distinct clade, the monophyly of the B genome clade cannot be assertively concluded due to the limited number of markers used in this study. However, the species *M. ornata* of the section *Rhodochlamys* was observed to be closer to *M. acuminata* than it was to *M. balbisiana*. This observation is consistent with the observation by Christelová etal. (2011) on *Musa* species where B genome is shown to have diverged 27.9 Ma while *M. ornata of* the section *Rhodochlamys* diverged only 8.8 Ma. Furthermore, the results that *M. ornata of* the section *Rhodochlamys* and *M. acuminata* of *Eumusa* being similar confirm Simmonds (1962) work which showed that hybridization between species of section *Eumusa* and *Rhodochlamys* are successful and attributed to lack of any reproductive barriers between the two sections.

Surprisingly in this work, the clustering of the ABB genotypes Pelipita, Monthan, Dole, and Saba to the B genome clade is contrary to what was observed by Boonruangrod etal. (2008). Indeed, clustering of the genotypes with ABB genome was not limited to the four genotypes but to all the ABBs; genotypes Kluai Tiparot and Namwa Khom were also observed to cluster to the B genome clade (figs. 3 and 4). The segregation of AAB genotypes is in accordance with the results obtained by Carreel etal. (2002), where all the AAB apart from Pisang Rajah and Pisang Kelat subgroups were found to have A genome cytoplasmic constitution. The segregation of this genomic group with *NAD1* was not only observed in the sequences but also in the sizes of the amplicons. This suggests that the mtDNA region used in this study may not solely and conclusively identify the mitochondrial types hence more markers may be needed.

The segregation based on the marker *CENH3* was similar in many ways to that of *NAD1*. The two major clades representing the A and B genomes observed in *NAD1* phylogeny were also observed with *CENH3*, which shows consistency. The identification of A and B was derived by the observation that no diploid BB or AA genotypes clustered together, they were in either one of the two main clades for both markers (fig. 4). The major deviation to the *NAD1* phylogeny was mainly in the A genome clade where this marker was unable to differentiate the subspecies (fig. 4). The marker *CENH3* is a nucleus based marker and hence the clustering of the A genome subspecies was expected.

The segregation of the A genome in subspecies *malaccensis* and *truncata* in this study compares favorably with the one observed by Wong (2002) where the two subspecies clustered separately. However, this observation deviates from the placement of the subspecies *truncata* as a synonym of the subspecies *malaccensis* by Hotta (1989). This study clearly differentiated the two subspecies, with *malaccensis* being supported as a separate clade from *truncata* (fig. 4). Despite the limited number of markers used in this study, our study confirms the closeness of the subspecies *burmannica* (Long Tavoy) and *burmannicoides* (Calcutta 4) similar to observations made by Ude etal. (2002) and later confirmed and proposed to be joined as one (Sardos etal. 2016; fig. 4).

The segregation within the B genome clade was consistent in both markers used in this study and is similar in some aspect to observations made by Ude etal. (2002), which observed that ABB genotypes were closer to BB diploids. In this study using both markers, the AAB and ABB genotypes were observed to segregate into either A or B genome clades. Similarly, Ude etal. (2002) observed a close clustering between the triploid AAB and ABB with the diploid AA and BB, respectively. However, AAB and ABB genomic groups were observed to have A and B as well as B and A haplotypes respectively (table 3).

Different studies have indicated that only *M. acuminata* subspecies that originated from the islands of Southeast Asia (ISEA) have had genomic contribution to cultivated bananas (Li etal. 2013; Perrier etal. 2011). In this study, both phylogeny and haplotype inference indicated that ISEA subspecies contributed to triploid cultivated bananas (figs. 3 and 4). Phylogeny using both *NAD1* and *CENH3* was able to establish one major clade (Clade A) that had ISEA subspecies *zebrina*, *errans*, *banksii*, *truncata*, and *malaccensis* clustering. Moreover, the EAHB have been shown to have had genomic contribution from ISEA subspecies *zebrina* and *banksii* (Perrier etal. 2011). In this study, based on *CENH3* marker, EAHB genotypes Ngombe and Mbwazirume were observed to cluster within clade A to major ISEA subspecies (figs. 3 and 4). These observations further attest to the ISEA subspecies contributing to EAHB. Observation of the diploid genotype Tomolo clustering with the wild type genotype *M. acuminata* subsp. *malaccensis* (fig. 4) was contrary to other observations made that have linked this cooking diploid cultivar to wild type subspecies *banksii* (Carreel etal. 2002; De Langhe etal. 2010; Li etal. 2013). However, this deviation was observed only with the phylogeny based on *CENH3* marker. This deviation can be explained by the fact that *CENH3* marker is a nuclear gene, while *NAD1* marker is of mitochondrial nature and the latter has uniparental inheritance.

The maternal and paternal nature of inheritance of chloroplast (cpDNA) and mitochondrial (mtDNA) genetic material in bananas (Fauré etal. 1993; Heslop-Harrison and Schwarzacher 2007) can facilitate the identification of the wild genotypes that contributed to banana cultivars. To infer haplotypes from amplified sequences, only the marker *CENH3* was used, due to the fact that extranuclear DNA (*NAD1*) in bananas is monoparentally inherited and no crossing over in the case of *NAD1* may have taken place during breeding. Furthermore, no multiple peaks were observed in the sequences obtained from triploid and diploid genotypes based on *NAD1*.

A total of 18 haplotypes (denoted by the number 1–18) were observed in this study. Haplotypes represented in a cultivar have been used as an indicator of the species that contributed to the cultivar and thus indicating the domestication patterns (Boonruangrod etal. 2008; Li etal. 2013). In our study, Haplotype 1 mainly consisted of B genome derived haplotypes (table 4). The two haplotypes for the genotype Kunnan (AB) were found to closely associate with haplotype 1, suggesting that indeed the B genome diploids contributed to these genotypes. Our results do not support the results obtained by Li etal. (2013), which suggested that this genotype should be renamed to AA. This observation however raises the question of why the A genome in the AB genotype was not identified in the analysis. The triploid genotypes Lady Finger (AAB), Saba (ABB), and Dole (ABB) were observed to have a haplotype similar to haplotype 1 which is a B genome haplotype (table 4). This indicates that B genome diploids contributed to the genomes of triploids and is in line with observations made from other studies (Li etal. 2013; Perrier etal. 2011). However, this study could not identify which BB diploid specifically contributed to the B genome in triploids. The clustering of two Lady Finger haplotypes to haplotype 2; which is essentially an A genome haplotype group, indicates that both AA haplotypes in the AAB haplotype of Lady Finger may have originated from the same *M. acuminata* subspecies. The two haplotypes of the genotype Wompa were related to Haplotypes 3 and 6. Haplotypes 3 had other genotypes of A genomic composition but no diploid wild type genotypes were in this haplotype. Two of the four genotypes that were together with Wompa in this haplotype were EAHB genotypes Ngombe and Mbwazirume. The other genotype with a haplotype represented in this group was the diploid genotype Tomolo (table 4). This indicates that Tomolo may have contributed its genome to EAHB genotypes. The approach used here provides a quick and easy approach to identify haplotypes and constitutive subgenomic polymorphisms.

In this study, no single genotype (either homogenomic or heterogenomic triploid) was observed to have mtDNA haplotypes that were obtained with the marker *NAD1*. This observation can be explained by the uniparental nature of mitochondrial inheritance in banana where both mitochondria and chloroplast DNA have previously been used to identify the origins of genomes in diploid, homo- and heterogenomic banana genotypes (Boonruangrod etal. 2008; Carreel etal. 2002). In the present study, direct sequencing of PCR products allowed to differentiate easily the maternal mtDNA types. We observed that multiple *CENH3* alleles may exist in bananas similar to observations made in other crops.

## Supplementary Material

Supplementary Data
