# Supplementary material for: Dominant Allele Phylogeny and Constitutive Subgenome Haplotype Inference in Bananas Using Mitochondrial and Nuclear Markers
Source: Genome Biol Evol. 2017 Aug 27;9(10):2510–21. doi: 10.1093/gbe/evx167 (PMC5629815; doi:10.1093/gbe/evx167)
Supplement: Supplementary Data [file evx167_supp.zip › Revised Supplementry Document.pdf]

Supplementary Figure

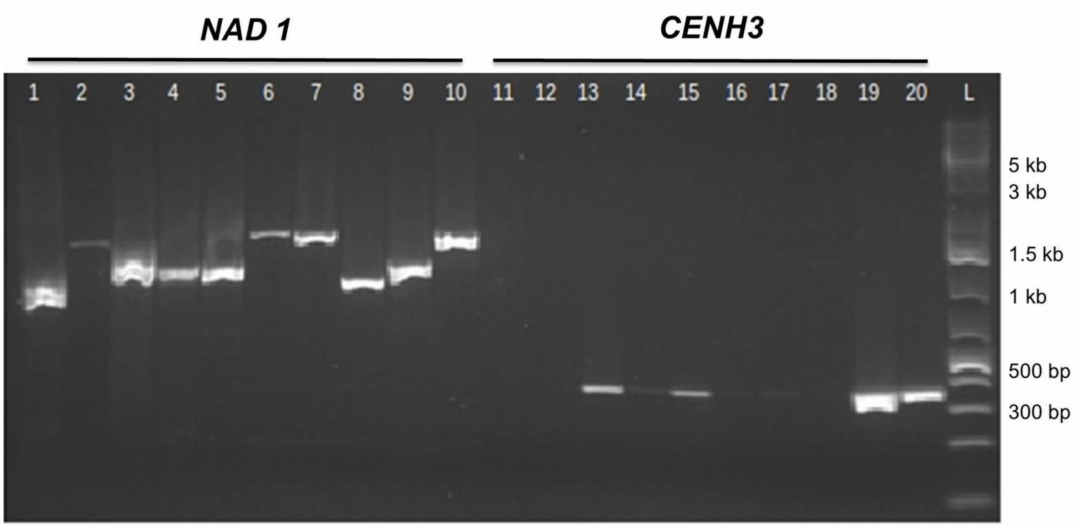

Supplementary Figure 1: PCR amplification of banana genotypes with different genomic composition using *NAD1* and *CENH3* specific primers. 1- AAB, 2, 3, and 10 - BB, 4 and 5 - AA, 6 and 7 - ABB, 8 *M. textilis* and 9 - *M. ornata*. Lanes 11 to 20 are a repetition of samples 1 to 10 amplified with the marker *CENH3*. L is 1 kb plus DNA ladder (Thermoscientific, MA, USA).

Supplementary Tables

Supplementary Table 1: Accession numbers of *NAD1* sequences from 27 monocot species from which *NAD1* primers were designed

Serial number	NCBI gene loci/reference number	Name	Maximum alignment length
1	DQ091229	nad1 int2R	1,220
2	DQ091230	nad1 exon 3	>1540
3	DQ143135	nad1 int2R	924
4	DQ143136	nad1 int2R	925
5	DQ143137	nad1 int2R	925
6	DQ143138	nad1 int2R	925
7	DQ143139	nad1 int2R	925
8	DQ143140	nad1 int2R	925
9	DQ143141	nad1 gene	>1049
10	DQ143142	nad1 gene	>1049
11	DQ143143	nad1 int2R	925
12	DQ143144	nad1 int2R	925

13	DQ143145	nad1 int2R	925
14	DQ143146	nad1 int2R	925
15	DQ143147	nad1 int2R	925
16	DQ786093	NAD1 intron 2	>1391
17	DQ786117	NAD1 intron 2	>492
18	DQ786118	NAD1 intron 2	1,399
19	DQ786156	NAD1 exon 3	>1522
20	DQ786158	NAD1 exon 3	>1576
21	EF151272	nad1 gene	>1392
22	EF151273	nad1 intron 2	>1393
23	EF151281	nad1 gene	>1392
24	EF221846	nad1 gene	>862
25	GQ265090	NAD1 intron 2	>1308
26	HM034957	nad1 gene	>1521
27	JQ350907	nad1 intron 2	>862

Supplementary Table 2: SNP positions that were used in inferring the haplotypes of triploid and diploid genotypes, the 'SNP positions in alignment' columns indicate the position of the *CENH3* sequence alignment that was used in haplotype inference.

			SNP positions in alignment						
Cultivar Name	Genome	Ploidy	62	63	95	120	122	166	208
Calcutta 4	AA	2N	CN	GN	AN	AN	AN	TN	CN
Malaccensis	AA	2N	CN	GN	AN	AN	AN	TN	CN
Agutay	AA	2N	CN	GN	AN	AN	AN	TN	CN
Long Tavoy	AA	2N	CN	GN	AN	AN	AN	TN	CN
Banksii	AA	2N	CN	GN	AN	AN	AN	TN	CN
Truncata	AA	2N	CN	GN	AN	AN	AN	TN	CN
Khae Phrae	AA	2N	CN	GN	AN	AN	AN	TN	CN
DH Pahang	AA	2N	CN	GN	AN	AN	AN	TN	CN
Tomolo	AA	2N	CN	GN	AG	AN	AG	TN	CN
Pisang Mas	AA	2N	NN	NN	NN	TN	AN	TN	CN
Zebrina	AA	2N	NN	NN	NN	AN	AG	CT	CN
Kunnan	AB	2N	CN	GN	AN	AN	AN	CN	TN
Safetvelchi	BB	2N	GC	GC	AG	AN	AN	TN	CN
Wompa	AS	2N	CN	GN	AN	AN	GN	TN	CT
Pisang Batu	BB	2N	CN	GN	AN	AN	AN	CN	TN
Honduras	BB	2N	CN	GN	AN	AN	AN	CN	TN
Pisang Klutuk Walung	BB	2N	CN	GN	AN	AN	AN	CN	TN
Lal Velchi	AB	2N	CN	GN	AN	AN	AN	CN	TN
Grande Naine	AAA	3N	CNN	GNN	ANN	ANN	ANN	TNN	CNN
Gombe	AAA	3N	CNN	GNN	ANN	ANN	GNN	TNN	CNN
Mbwazirume	AAA	3N	CNN	GNN	ANN	ANN	GNN	TNN	CNN
Pisang Kayu	AAA	3N	CNN	GNN	ANN	ATN	ANN	TNN	CNN
Figue Pomme Geante	AAB	3N	CNN	GNN	ANN	ANN	ANN	TNN	CNN
Pisang Ceylan	AAB	3N	CNN	GNN	GNN	ANN	AGN	TNN	CNN
Prata Ana	AAB	3N	GCN	GCN	AGN	ANN	ANN	TNN	CTN
Orishele	AAB	3N	CNN	GNN	ANN	ANN	ANN	TNN	CNN
Foconah	AAB	3N	CNN	GNN	ANN	ANN	ANN	TNN	CNN
Lady Finger	AAB	3N	CNN	GNN	ANN	ANN	ANN	CTN	CTN
Red yade	AAB	3N	NNN	NNN	NNN	NNN	TNN	CNN	GNN
Pelipita	ABB	3N	CNN	GNN	ANN	ANN	ANN	TNN	CNN
Namwa Khom	ABB	3N	GNN	GNN	GNN	ANN	ANN	TNN	CTN
Dole	ABB	3N	CNN	GNN	ANN	ANN	ANN	CNN	TNN
Saba	ABB	3N	CNN	GNN	ANN	ANN	ANN	CNN	TNN
Kluai Tiparot	ABB	3N	CNN	GNN	ANN	ANN	ANN	CNN	TNN

Supplementary Table 3: Model selection for the marker *NAD1*

Model	Decoration	K	Llk	AIC	BIC			
GTR		79	-1582	83880	3323	67760	3674	63827
TN93		76	-1592	92583	3337	85166	3675	48471
GTR	+G	80	-1580	94107	3321	88214	3677	28535
GTR	+I	80	-1581	20185	3322	40370	3677	80691
GTR	+G+I	81	-1580	95394	3323	90788	3683	75363

Supplementary Table 4: Model selection for the marker *CENH3*

Model	Decoration	K	Llk	AIC	BIC			
TN93	+G	75	-452	37732	1054	75464	1315	48940
TN93		74	-456	31006	1060	62012	1317	87842
TN93	+I	75	-453	76492	1057	52984	1318	26460
HKY85	+G	74	-457	59	1062	118	1319	25948
TN93	+G+I	76	-452	37726	1056	75452	1320	96574
GTR	+G	78	-447	46397	1050	92794	1322	9209
GTR	+I	78	-448	85666	1053	71332	1324	87747
GTR		77	-451	82668	1057	65336	1325	34105
GTR	+G+I	79	-447	44720	1052	89440	1327	53502

Legends for Supplementary Data

Supplementary Data 1: Phylogenetic Tree for the marker *NAD1* in Newick format

Supplementary Data 2: Phylogenetic Tree for the marker *CENH3* in Newick format
